# A Comparative Analyses of Granulometry, Mineral Composition and Major and Trace Element Concentrations in Soils Commonly Ingested by Humans

**DOI:** 10.3390/ijerph120808933

**Published:** 2015-07-31

**Authors:** Veronica M. Ngole-Jeme, Georges-Ivo E. Ekosse

**Affiliations:** 1 Department of Crop Science, Faculty of Agriculture, Science and Technology, North West University, Private Bag X2046, Mmabatho 2735, South Africa; 2 Directorate of Research and Innovation, University of Venda, Private Bag X5050, Thohoyandou, Limpopo Province 0950, South Africa; E-Mail: ekosseg@gmail.com

**Keywords:** geophagia, primary minerals, secondary minerals, chemical index of alteration, specific surface area/volume, Sauter Mean Diameter

## Abstract

This study compared the granulometric properties, mineralogical composition and concentrations of major and trace element oxides of commonly ingested soils (geophagic soil) collected from different countries with a view of understanding how varied they may be in these properties and to understand the possible health implications of ingesting them. Soil samples were collected from three different countries (South Africa, Swaziland and Democratic Republic of Congo (DRC)) and their granulometric properties, concentrations of major and trace element oxides as well as mineralogical composition determined. Differences were observed in the granulometric properties of geophagic soil from the three different countries with most of them having <20% clay content. The soils also showed varied degrees of weathering with values of Chemical Index of Alteration (CIA) and Chemical Index of Weathering (CIW) being between 60% and 99.9% respectively. The mineral assemblages of the soils from South Africa and Swaziland were dominated by the primary minerals quartz and feldspar whereas soils from DRC had more of kaolinite, a secondary mineral than primary minerals. Soils from DRC were associated with silt, clay, Al_2_O_3_, and CIA unlike most samples from South Africa which were associated with SiO_2_, sand, K_2_O, CaO, and MgO. The soils from Swaziland were closely associated with silt, H_2_O and Fe_2_O_3_(t). These associations reflect the mineralogy of the samples. These soils are not likely to serve as nutrient supplements because of the low concentrations of the nutrient elements contained. The coarse texture of the samples may also result in dental destruction during mastication. Sieving of the soils before ingestion to remove coarse particles is recommended to reduce the potential health threat associated with the ingestion of coarse-textured soils.

## 1. Introduction

Around the globe deliberate ingestion of soil (geophagia), especially among women, is a habit that has been categorized as a medical condition by the World Health Organisation [1]. Despite the negative connotations that have been ascribed to it, geophagia still remains very widespread and has no boundaries with regards to race, socio-economic status, religious orientation or ethnic origin [2]. A variety of soil and clay types are deliberately ingested by humans and other animals around the globe for three main reasons; dietary deficiencies, plant toxins, or gastrointestinal distress [3]. These soils are collected from different sources which include but are not limited to termite mounds, brick walls, open fields, river beds, mountains, and road sides [4]. The specific reasons for soil ingestion include; adjustment of imbalance or deficiency of minerals, pH adjustment in the digestive system, treatment of ailments, food detoxification, and alleviation of gastrointestinal disorders, such as diarrhoea [5,6,7,8,9,10,11,12,13], and protection from aflatoxins [14,15]. While ingesting soils may be beneficial for these purposes, geophagia has also been implicated in several health complications.

Though soil has been ingested to alleviate symptoms of Fe deficiency anemia, Severance *et al.* [16] and Hooda and Henry [17] have shown that geophagia has in some cases caused Fe deficiency. This was attributed to the cation exchange capacity (CEC) of the ingested soils [18]. Geophagia is also reported to play a significant role in the prevalence of soil transmitted helminth infections in several communities [19,20,21]. Surface soils may contain external elements such as feces causing diarrhea, helminth infections, or just plain poisoning [22]. Chemical analyses of geophagic soils have revealed the presence of harmful components including heavy metals and organic compounds which may adversely affect individuals ingesting such soils. Ingesting soils with high concentrations of useful components like iron according to Henry and Cring [3] may also result in health complications like hemosiderosis. Coarse sand-sized quartz particles in geophagic soils could affect dental enamel and the sigmoid colon due to the abrasive nature of the particles.

The ability of the geophagic soil to fulfill the purpose for which they are ingested is influenced by the properties of the soils. Soil physical properties, and mineralogical and chemical compositions dictate their behavior and influence the interactions that occur between the ingested soil and other contents in the gastrointestinal tract (GIT) of the individual ingesting them. The significance of physico-chemistry, and mineralogy of soils ingested by humans have been highlighted in Henry and Cring [3], Hooda [10] Hooda and Henry [17], Seim *et al.* [23], Pebsworth *et al.* [24], Ekosse [25], Ngole and Ekosse [26], Abraham [27], and Mahaney *et al.* [28]. There have been studies by Dominy *et al.* [12], Pebsorth *et al.* [24], and Kutalek *et al.* [29] to determine the chemical properties of geophagic soils and the bioaccesibility of the nutrients they may contain in the GIT. Other studies have focused on the mineralogical composition of these soils [25,26] and reasons why individuals ingest them [2,11,30,31].

Granulometric properties of soils describe their particle size characteristics and play an important role in several soil reactions. Particle size distribution (PSD) of soils could provide clues to possible health threats that the habit of geophagia may present to an individual. King *et al*. [32] have shown that ingestion of coarse textured soils causes severe dental damage in hominid species. Alleviation of gastrointestinal upsets and detoxification of noxious or unpalatable compounds present in the diet of individuals have been presented as a reason to justify geophagia among humans [11]. This function is linked to the surface area of ingested soils which depend on the PSD of the soils. All sorption reactions in soils are related to their cation exchange capacity (CEC), which is determined by the texture and the mineral assemblage of the soil. Deficiencies in certain elements like Ca and Fe have also been used to justify geophagic habits especially in pregnant and lactating mothers [33,34] but the release of these nutrients from ingested soil into the GIT of the individual ingesting the soil and their assimilation depends on several aspects including the CEC of the soil, chemical environment in the GIT, as well as other contents in the GIT. Supplementation of Ca and Fe will also depend on the concentrations of these elements in the soil which to a large extent depends on the type of soil and the degree of weathering that has taken place. Studies carried out by Pebsworth *et al.* [24] revealed that high content of Fe in geophagic soils does not necessarily translate into high bioavailability. Soil mineralogy plays an important role in the behavior of soils and is therefore an important soil property to include in the characterization of geophagic soils [11].

Studies which have focused on the ganulometric characteristics of soils commonly ingested by humans are scarce. Comparative studies aimed at understanding the granulometry, mineralogy and oxide chemistry of geophagic soils are also lacking. This study compares the granulometric properties, mineralogical composition and concentrations of major and trace element oxides of geophagic soils collected from different countries (Swaziland, South Africa and Democratic Republic of Congo) with a view of understanding how varied geophagic soils ingested by humans may be in these properties and the potential implications of these properties on the health of individuals ingesting them. It also addresses the extent of weathering of the soils and possible association between granulometric properties, samples and selected chemical properties using a multivariate approach.

## 2. Description of Soils in the Study Area

**Figure 1:**
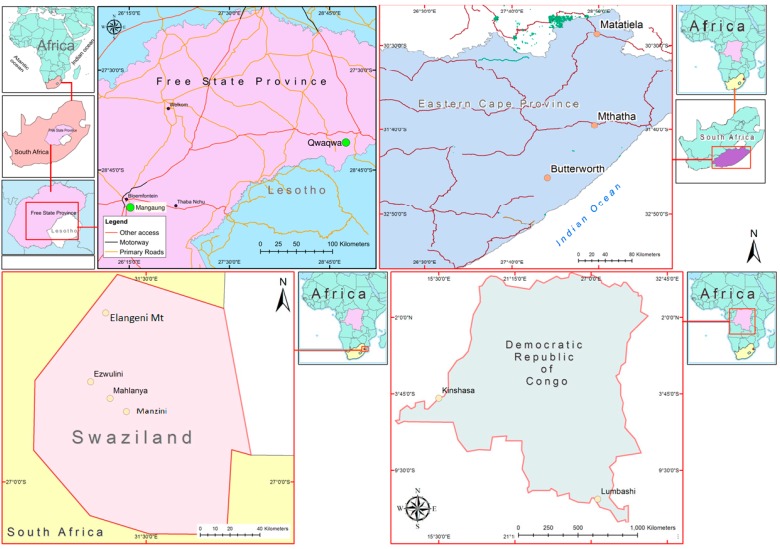
Map showing location of sampling points in the three different countries.

Soils ingested in Swaziland were collected from Ezulwini, Mahlanya, Mphembekati and Mbekelweni (Manzini neighborhood), Elangeni Mountain, and Nsingweni (Figure 1). Swaziland soils are highly weathered ferralitic soils having a low cation exchange capacity (CEC) in the high veld region whereas those from the low veld region (Manzini, Mahlanya and Ezulwini) are moderately weathered with a higher clay content and CEC [35]. The soil samples from Democratic Republic of Congo were collected from Lubumbashi and Kinshasa. Though arenosols derived from the Kalahari sands dominate the soil cover of Kinshasa, strongly weathered ferralsols make up a greater part of the soil of DRC [36]. These soils are characterised by a sandy texture, low clay content and low CEC. Samples of geophagic soils ingested in South Africa came from Bloemfontein and the Eastern Cape. Sandy to clayey and shallow soils are found in the Bloemfontein region whereas soils from the Eastern Cape generally contain less than 15% clay and are mainly derived from the weathering of basalt and dolerite origin. Quartz, mica, and/or kaolinite dominate the mineral composition of these soils [37].

## 3. Experimental Section

### 3.1. Collection of Geophagic Soil Samples

Whereas some of these soils (EN4, ECK15, FSB19, FSB26, SZ32, SZ34, SZ35, DRC1, DRC5, DRC6, DRC7, DRC8, DRC10, DRC12, DRC13, and DRC15) were bought from vendors, the others were collected from identified geophagic mining sites. The soils were collected from river beds, traditional clay mines or village ponds that had dried off using a hand trowel. Information on the sources of the various soils samples and the methods of processing was obtained from the vendors from whom the samples were bought. The color of the soils was determined with the Munsell color chart and the colors presented using hue, value and chroma. The hue refers to the color shade of the soil on the electromagnetic spectrum (R = red, Y = yellow and YR = yellow red). The higher the hue, the purer the color of the soil. The value of a soil color indicates the amount of light reflected where 10 = total reflection (white soil) and 0 = no light reflected (black soil). Chroma of a soil refers to the concentration of the hue. Colors of low chroma are referred to as weak while those of high chroma are said to be highly saturated, strong or vivid.

### 3.2. Determination of Granulometric Properties of Geophagic Soils

Particle size distribution of the soils was determined by laser light scattering using a Malvern Masteriser 2000 particle size analyzer. Each sample was treated with sodium hexametaphosphate (Na_4_P_2_O_7_), 30% H_2_O_2_, and 10% HCl as described in van Reeuwijk [38], Ngole and Ekosse [26] and Council for Geosciences [39] to disperse the samples. A suspension of each sample was then loaded into a Malvern Mastersizer 2000 fitted with a Hydro 2000G dispersion unit. A polydisperse mode of analysis and a refractive index of 1.53 with an adsorption of 0.1 were chosen. Size data collection was performed at constant obscuration in the range 10–20%. Scattered light data were recorded from 2000 to 5000 snapshots of 10 *μ*s. The correlation between the angles of light scattered from the particles in a laser beam was used to determine the size distribution of these particles. Particle data generated for each soil sample included the PSD and derived diameters (D(v,0.1), D(v,0.5), D(v,0.9), and D[3,2]), where D(v,0.1), D(v,0.5) and D(v,0.9) represent the 10^th^ percentile, median diameter, and 90^th^ percentiles respectively of the soil samples. Values for D(v,0.1) and D(v,0.9) describe the maximum particle diameter below which 10% and 90% respectively of each sample volume exist. D[3, 4] describes the Sauter Mean Diameter (SMD) which is the diameter of a particle having the same surface area: Volume ratio as that of the entire sample. The specific surface area volume ratios (SSA/V) of the samples were calculated using particle size data and SMD. Samples were run in duplicate with three runs per replicate. The weight percent (wt%) of sand, silt and clay was plotted in a textural triangle to determine the texture of the samples.

### 3.3. Mineralogical Analyses of the Soil Samples

The mineralogy of the bulk soil was determined using an X-ray diffractometer (XRD) as described by Council for Geosciences [39], Brime [40], Bish and Reynolds [41], and Moore and Reynolds [42]. Both qualitative and semi-quantitative mineralogical analyses were performed on each soil sample. Each sample was crushed, milled and homogenized to a fine powder of approximately 10–15 *µ*m in size. A subsample was then pressed into shallow sample holders against a rough filter paper to ensure random orientation [39]. Each sample was then scanned from 2° 2θ to 70° 2θ with CuKα radiation (40 kV and 40 mA), using a 0.02° 2θ scanning step and 0.5 s counting time per step, with a LYNXEYE detector. Minerals contained in each sample were identified using the ICDD 2004 database of minerals. Phase concentrations were determined as semi quantitative estimates, using relative peak heights/areas proportions and reference-intensity-ratio (RIR) method [40].

### 3.4. Determination of Major and Trace Element Oxides in Soil Samples

The concentrations of major and trace element oxides in each of the sample was determined using X-ray Fluorescence (XRF). Major and trace element oxides determined in soil samples included SiO_2_, TiO_2_, Al_2_O_3_, Fe_2_O_3_(t), MnO, MgO, CaO, Na_2_O, K_2_O, P_2_O_5_ and Cr_2_O_3_. A samples of each soil was ground to a grain size of below 75 μm and calcined at 1000 °C for 3 h. One gram of calcined sample and 9 g of flux consisting of 34% LiBO_2_ and 66% Li_2_B_4_O_7_ were fused at 1050 °C to form stable glass beads which were then analyzed for major and trace element oxides using A PANalytical Axios WDXRF spectrometer. Details of the method have been described by the Council for Geosciences [39], and Fitton [43]. A secondary amphibolite reference material was used as a quality assurance sample to ascertain the accuracy of the results generated by the equipment. To determine the extent of weathering of the samples, the Chemical Index of Alteration (CIA, [Al_2_O_3_/(Al_2_O_3_ + CaO + Na_2_O + K_2_O)] × 100) as well as the Chemical Index of Weathering (CIW) (where CIW = [Al2O3/(Al2O3 + CaO + Na2O)] × 100) of each sample was calculated [44,45].

### 3.5. Statistical Analyses

All analyses were carried out in duplicate. A general linear model (GLM) was used to separate means of the three different countries using IBM Statistical package for Social Sciences (SPSS 22). Each mean value is presented with the standard errors of the mean of each parameter. All comparisons were carried out at 95% confidence limit. An RQ mode of Principal Component Analyses (PCA) as described by Zhou *et al.* [46] was used to calculate variables and object loadings on the same axis. A biplot was then used to facilitate recognition of subgroups and determining the basis of group separation. It was also used to understand how the samples from the three different countries differed.

## 4. Results

### 4.1. Description of Soil Samples

The color of the soils varied from whitish to greyish, yellowish brownish and reddish or different color combinations of the above. A majority of the soils had a hue of 10R, 10YR, 2.5Y and 2.5YR. The color value of the soils were 4, 5, 7 and 8, and chroma of 1, 4 and 8. Finger assessment of the soil samples after collection indicated that some of the soils especially those from South Africa and Swaziland felt gritty whereas majority of the soils from DRC felt clayey to the touch. Some of the soils that were bought from vendors were processed in different ways; salt was added to some of them (DRC1 and DRC4) and then molded into ball-like shapes before selling. Soils bought in Swaziland (SZ32, SZ34, SZ35), were molded without adding any salt. Some of the soils from DRC were molded and either burnt or baked to improve their organoleptic properties and possibly reduce pathogen load. The processing of samples was carried out by the vendors prior to selling.

### 4.2. Granulometric Properties of Soil Samples

#### Particle Size Characteristics

Values for the median diameter of the particles making up the soils ranged from 7 µm–132 µm in samples from South Africa, 8 µm–71 µm in samples from Swaziland and 0.5 µm–28 µm in samples from DRC (Table 1) with mean median diameters of 46.3 µm ± 6.0, 27.6 µm ± 8.1 and 6.61 µm ± 7.3 for samples from South Africa, Swaziland and DRC respectively. Mean value for derived diameters D(v,0.1) and D[3,2] were higher in samples from South Africa (6.7 µm and 12.0 µm) compared to those from Swaziland (3.9 µm and 8.0 µm) and DRC (1.2 µm and 2.9 µm).

**Table 1:** Granulometric characteristics of geophagic soils.

Country from Where Samples Were Collected	Sampling Site	Sample I.D.	Specific Surface Area/Volume (micron^-1^)	Sand ^*^ (%)	Clay ^*^ (%)	Silt ^*^ (%)	Derived Diameters (*µ*m)			
							D(v,0.1)	D(v,0.5)	D(v,0.9)	D[3,2]
South Africa	Eastern Cape	EN1	0.25	73	2	25	17,62	124,41	296,79	23,74
		EN4	0.32	70	2	28	11,21	99,41	449,58	18,87
		ECM6	0.54	51	4	44	4,89	52,53	201,12	10,64
		ECM7	0.57	59	4	36	5,42	86,11	330,27	11,40
		ECM8	1.74	5	14	80	1,41	10,74	43,96	3,53
		ECK9	0.07	88	0	12	43,32	132,94	369,51	70,74
		ECK10	1.10	24	10	66	2,12	20,47	104,53	5,21
		ECK12	1.13	21	10	69	1,96	14,75	79,70	4,89
		ECK13	0.33	69	3	28	10,70	91,41	252,14	16,69
		ECK14	1.82	12	17	70	1,35	8,44	67,40	3,52
		ECK15	1.16	18	10	72	2,00	20,31	70,92	5,17
		ECK16	0.57	54	4	41	5,08	60,61	290,66	11,05
		ECK17	0.39	57	3	40	9,91	64,59	308,75	15,78
		FSB18	1.39	11	13	76	1,68	12,98	54,76	4,43
	Bloemfontein	FSB19	1.10	12	9	79	2,10	17,69	54,55	5,40
		FSB20	1.01	11	8	80	2,59	21,21	54,47	6,20
		FSB21	1.57	9	15	76	1,42	9,32	46,62	3,68
		FSB22	0.56	47	4	49	5,58	45,96	238,90	11,25
		FSB23	0.80	17	6	77	3,27	25,15	59,39	7,21
		FSB25	0.38	60	3	37	7,50	62,05	218,64	13,94
		FSB26	2.18	6	18	76	1,13	7,03	35,69	2,78
	Eastern Cape	ECB27	0.69	43	5	52	3,74	31,20	279,44	8,11
Swaziland	Mahlanya	SZ28	1.19	18	9	73	2,19	16,90	155,30	5,11
	Manzini	SZ29	0.73	32	5	62	4,19	31,39	284,39	7,88
		SZ30	1.40	12	12	76	1,61	12,64	55,77	3,98
	Nsingweni	SZ31	0.50	38	3	58	5,46	37,27	371,22	11,17
	Ezulwini	SZ32	1.02	12	7	81	2,72	16,11	54,90	5,81
	Manzini	SZ33	0.32	62	2	36	8,27	71,74	687,35	16,86
	Nsingweni	SZ34	0.42	50	2	47	6,55	49,18	405,35	13,58
	Elangeni Mt	SZ35	0.98	22	8	70	3,12	23,13	313,14	6,49
	Nsingweni	SZ36	1.4	15	10	75	1,89	16,12	67,90	4,28
		SZ37	1.7	4	15	81	1,40	8,86	34,54	3,49
	Elangeni Mt	SZ38	0.5	31	3	66	7,11	31,75	129,90	11,62
	Manzini	SZ39	1.06	12	8	80	2,52	16,59	53,86	5,32
DRC	Kinshasa	DRC-1	1	5	4	91	2,62	7,55	25,14	5,69
		DRC-2	1.15	4	5	91	2,48	6,77	26,74	5,30
		DRC-3	21.9	2	57	41	0,10	0,75	13,33	0,26
		DRC-4	1.1	1	4	95	2,54	6,97	18,86	5,31
		DRC-5	16.79	7	45	49	0,11	3,57	36,46	0,35
		DRC-6	0.61	35	3	62	3,39	28,93	172,37	10,13
		DRC-7	15.14	7	42	51	0,11	3,39	38,54	0,35
		DRC-8	3.67	5	25	70	0,17	5,36	34,78	0,64
	Lubumbashi	DRC-9	19.1	1	59	40	0,09	0,46	14,44	0,25
		DRC-10	9.87	0	26	74	0,15	7,12	21,48	0,56
		DRC-11	0.95	0	2	98	2,88	8,18	25,10	6,16
		DRC-12	17.9	2	56	41	0,10	0,46	22,26	0,26
		DRC-13	0.7	2	3	95	3,36	14,50	35,48	8,30
		DRC-14	15.85	3	51	47	0,10	1,99	15,91	0,30
		DRC-15	5.1	1	39	60	0,12	3,11	17,54	0,37

^*^ The diameter of sand silt and clay are: Clay = < 2 µm, silt = < 50 µm–2 µm, and Sand = < 2000 µm–50 µm.

**Figure 2:**
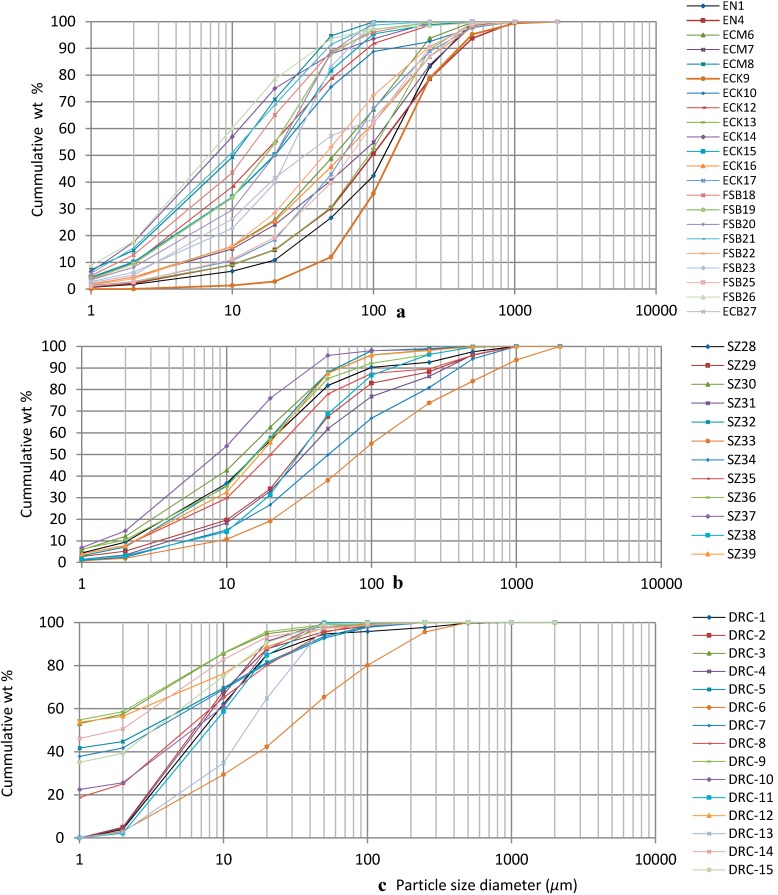
Particle size distribution curve of geophagic samples from South Africa (**a**), Swaziland (**b**), and DRC (**c**).

The derived diameters of individual samples are presented in Table 1. Samples from Swaziland had higher mean values of D(v,0.9) (214.8 µm) compared with 177.6 µm and 34.6 µm for samples from South Africa and DRC, respectively. Values for SMD of the samples followed the order soil samples from South Africa > soil samples from Swaziland > soil samples from DRC. Geophagic soils from South Africa contained more sand particles (mean = 31.14% ± 2.8) than those from Swaziland (mean = 25.76% ± 3.8) and DRC (mean = 5.0% ± 3.4) (Figure 2).

The silt content was generally higher than the sand content in all soil samples with only 17 of the 49 (35 %) samples analyzed having silt-sized particles constituting <50% (Figure 2). Clay-sized particles in the soil samples from South Africa and Swaziland comprised <20% by volume of the particles contained in the geophagic soil (Figure 2). This was different from the geophagic soil from DRC where clay-sized particles comprised up to 55% by volume in some samples (Figure 2). The soil samples from South Africa generally had lower mean values for SSA/V (0.89 m^2^/m^3^ ± 1.0) compared to the samples from Swaziland (0.94 m^2^/m^3^ ± 1.3) and DRC (8.7 m^2^/m^3^ ± 1.1). However, these differences were only significant between samples from DRC and those from Swaziland and South Africa (*p* < 0.05). Details of SSA/V for all samples are presented in Table 1.

### 4.3. Major and Trace Element Oxide Concentrations of Soil Samples

Results of the total concentrations of major and trace element oxides showed an accuracy of between 77 %–113 % for all oxides according to results from the reference sample (Table 2). Oxides of Si, Al, and Fe made up the greatest percentage of the trace and major element oxides in geophagic soils from all three countries (Table 2). Mean values for SiO_2_ concentrations were significantly higher in soils from South Africa (70.3% ± 2.1) compared to those from Swaziland (55.1% ± 2.9) and DRC (53.7% ± 2.5). Results presented in Table 2 indicate that the concentration of aluminum oxide was lowest in soils from South Africa. The concentrations of major oxides of Ca, Mg, Na, were relatively low according to data in Table 2 especially in the soils from DRC where CaO and K_2_O had significantly lower values than soils from Swaziland and SA (*p* < 0.05).

**Table 2:** Total concentrations of major and trace element oxides in geophagic samples.

Country	Sample	Oxide Concentrations (Wt%)											(%)		
		SiO_2_	TiO_2_	Al_2_O_3_	Fe_2_O_3_(t)	MnO	MgO	CaO	Na_2_O	K_2_O	P_2_O_5_	Cr_2_O_3_	Total	CIA	CIW
**South Africa**	EN1	77.68	1.11	5.39	5.87	0.06	0.44	2.10	0.53	0.76	0.10	0.02	99.96	61.38	67.19
	EN4	74.14	1.01	11.13	4.77	0.05	0.61	0.17	0.37	1.31	0.04	0.01	100.15	85.76	95.38
	ECM6	77.17	0.51	10.03	3.34	0.04	0.46	1.13	1.58	1.15	0.08	0.01	100.43	72.27	78.77
	ECM7	90.34	0.51	4.77	1.84	0.05	0.20	0.34	0.40	0.87	0.04	0.01	101.98	74.76	86.54
	ECM8	42.76	1.41	26.48	17.20	0.05	0.48	0.03	0.02	0.39	0.04	0.04	100.15	98.34	99.81
	ECM9	71.71	1.78	8.96	6.86	0.14	1.41	2.53	1.05	0.92	0.10	0.03	100.43	66.59	71.47
	ECK10	63.69	0.72	18.55	7.08	0.07	1.29	0.03	0.10	2.74	0.06	0.01	100.32	86.58	99.29
	ECK12	66.81	0.80	14.09	9.55	0.05	0.81	0.21	0.38	1.68	0.08	0.02	100.32	86.11	95.98
	ECK13	86.09	0.38	4.64	2.42	0.05	0.17	0.24	0.79	0.96	0.07	0.01	100.34	70.01	81.85
	ECK14	72.23	0.93	16.71	2.65	0.01	0.38	<0.01	<0.01	2.29	0.04	0.01	100.22	87.85	99.85
	ECK15	71.41	1.05	10.00	7.29	0.06	0.63	0.28	0.28	1.35	0.41	0.02	99.81	83.99	94.71
	ECK16	75.14	0.85	8.72	3.92	0.08	0.58	0.85	0.92	1.27	0.12	0.02	100.62	74.13	83.12
	ECK17	83.38	1.39	5.72	4.11	0.05	0.29	0.11	0.23	0.69	0.03	0.01	100.64	84.66	94.37
	FSB18	73.87	0.97	15.86	1.80	0.00	0.48	0.02	0.22	2.33	0.03	0.01	100.17	86.05	98.51
	FSB19	66.39	0.84	18.60	4.73	0.01	0.63	<0.01	<0.10	3.20	0.06	0.01	100.22	84.88	99.40
	FSB20	68.55	0.76	17.44	4.02	0.03	1.08	<0.01	0.04	2.56	0.09	0.01	100.14	87.00	99.73
	FSB21	65.99	0.72	17.10	6.73	0.01	0.86	0.15	0.08	3.19	0.07	0.01	100.12	83.33	98.65
	FSB22	66.81	0.85	17.47	4.30	0.02	0.98	0.28	0.76	3.50	0.07	0.01	99.60	79.36	94.37
	FSB23	69.11	0.75	17.27	3.91	0.03	1.06	0.01	0.05	2.51	0.09	0.01	100.24	87.04	99.63
	FSB24	69.85	0.87	17.28	3.11	0.01	0.85	0.33	0.07	1.56	0.03	0.01	100.64	89.76	97.70
	FSB25	73.62	0.78	14.61	3.07	0.02	0.85	0.05	0.79	2.79	0.12	0.01	100.22	80.10	94.54
	FSB26	43.46	1.74	25.57	17.63	0.07	0.42	<0.01	0.01	0.15	0.05	0.03	100.16	99.34	99.94
	FSB27	66.60	0.87	15.05	5.57	0.06	1.51	0.89	2.30	1.64	0.03	0.02	99.60	75.73	82.52
**Swaziland**	SZ28	57.24	0.97	24.27	8.47	0.04	0.18	<0.01	<0.01	0.16	0.02	0.01	100.40	99.28	99.92
	SZ29	49.31	0.72	18.04	19.58	0.05	0.48	1.20	2.10	1.00	0.16	0.01	99.80	80.74	84.52
	SZ30	46.16	0.60	26.55	14.38	0.13	0.83	<0.01	0.03	0.35	0.03	0.03	100.08	98.56	99.85
	SZ31	59.33	0.51	25.02	2.90	0.03	0.53	<0.01	4.46	2.03	0.03	0.00	100.16	79.39	84.85
	SZ32	68.34	0.50	16.62	1.58	0.02	0.27	<0.01	0.02	1.34	0.02	0.00	100.11	92.38	99.81
	SZ33	43.10	1.57	28.09	20.82	0.08	0.24	<0.01	<0.01	0.11	0.03	0.03	100.02	99.53	99.93
	SZ34	55.21	0.09	29.15	1.65	0.01	0.49	<0.00	4.71	3.37	0.01	0.00	100.07	78.29	86.09
	SZ35	70.38	0.15	18.61	2.06	0.03	0.11	<0.01	0.02	3.83	0.02	0.01	100.41	82.81	99.83
	SZ36	48.23	0.03	27.78	12.18	0.00	0.03	<0.01	<0.01	0.05	0.05	0.04	100.11	99.74	99.93
	SZ37	45.74	0.03	38.57	0.84	0.00	0.25	<0.01	<0.01	0.74	0.05	0.01	99.81	98.08	99.95
	SZ38	72.07	<0.01	18.56	1.35	0.09	0.01	<0.01	0.09	2.88	0.02	0.01	100.33	86.13	99.44
	SZ39	46.27	1.58	24.92	15.13	0.07	0.26	<0.01	<0.01	0.12	0.05	0.01	99.99	99.43	99.92
**DRC**	DRC1	54.74	0.81	28.09	1.35	0.01	0.76	0.02	1.13	1.02	0.07	0.01	100.43	92.82	96.05
	DRC2	53.94	0.80	29.32	1.64	0.01	0.89	0.04	<0.01	1.19	0.08	0.01	100.26	95.92	99.82
	DRC3	46.52	1.25	32.55	6.48	0.02	0.16	<0.01	0.01	0.21	0.07	0.01	100.08	99.29	99.94
	DRC4	51.48	0.89	30.21	1.09	0.01	0.71	0.03	1.02	0.79	0.06	0.01	99.57	94.28	96.65
	DRC5	53.76	1.29	23.04	13.12	0.02	0.02	<0.01	<0.01	0.92	0.21	0.02	100.04	96.08	99.91
	DRC6	72.29	1.08	18.98	0.48	0.00	0.08	<0.01	<0.01	0.04	0.05	0.01	100.42	99.66	99.89
	DRC7	54.11	1.30	23.42	12.45	0.02	0.05	<0.01	<0.01	0.95	0.21	0.02	100.20	96.03	99.91
	DRC8	61.02	1.18	26.70	0.72	0.00	0.74	<0.01	<0.01	1.74	0.02	0.02	100.41	93.81	99.93
	DRC9	43.95	1.41	32.94	7.70	0.02	0.11	<0.01	<0.01	0.14	0.06	0.02	100.25	99.53	99.94
	DR10	60.91	1.43	22.13	4.50	0.00	1.00	<0.01	0.36	2.43	0.03	0.02	100.45	88.75	98.35
	DR11	60.47	0.07	0.65	0.40	0.02	32.64	0.02	<0.01	0.13	0.02	<0.001	98.99	80.63	95.75
	DR12	44.07	1.69	35.60	4.32	0.01	0.15	<0.01	<0.01	0.05	0.04	0.02	100.56	99.81	99.94
	DR13	53.92	2.57	27.25	3.76	0.01	0.61	0.04	<0.01	1.01	0.06	0.01	99.99	96.25	99.82
	DR14	46.46	1.31	32.97	6.41	0.02	0.18	<0.01	<0.01	0.23	0.08	0.01	100.56	99.26	99.94
	DR15	47.42	1.33	36.20	1.53	0.01	0.28	<0.01	<0.01	0.21	0.06	0.01	101.45	99.36	99.94
	Certified	45.42	1.54	16.62	9.73	0.18	8.15	10.93	3.65	0.70	0.26	0.07	99.75	52.10	53.27
	Results	44.34	1.55	16.44	9.76	0.18	8.29	11.10	3.31	0.68	0.22	0.07	98.79	52.13	53.28

### 4.4. Mineral Composition of the Soil Samples

The mineral assemblage of the soils from South Africa (Table 3) and DRC (Table 4) were more diverse than in those from Swaziland (Table 5). Minerals identified in the soil samples included both primary and secondary minerals. The primary minerals identified were quartz (SiO_2_), microcline (KAlSi_3_O_8_), anatase (Ti0_72_O_2_), muscovite K(Si_75_Al_25_)_4_Al_2_O_10_(OH)_2_), ilmenite (FeTiO_3_), magnetite (Fe_2.25_Ti0_75_O_4_), anorthite (Ca,Na)(Al,Si)_2_Si_2_O_8_), alunite (KA_l3_(SO_4_)_2_(OH)_6_, and serpentine (Mg_3_(OH)_4_(Si_3_O_5_). Secondary minerals identified included kaolinite (Al_2_Si_2_O_5_(OH)_4_), Calcite, (CaCO_3_), dolomite (CaMg(CO_3_)_2_, gibbsite (Al(OH)3), halite (NaCl), alunite (K(Al_3_(SO_4_)_2_OH)_6_, siderite (FeCO_3_), goethite (FeO(OH), hematite (Fe_2_O_3_), talc (Mg_3_(OH)2Si_4_O_10_), and smectite (Na_0.3_(Al,Mg)_2_Si_4_O_10_-(OH)_2_.xH_2_O). Primary soil minerals constituted a greater percentage of the mineral assemblage of most of the samples from South Africa (Table 3).

**Table 3:** Mineral assemblage of soil samples from South Africa.

Sample Code	Mineral Abundance (Wt%)										
	Magnetite	Anatase	Hematite/Goethite	Microcline/Rutile	Plagioclase	Quartz	Kaolinite/Chlorite	Kaolinite	Muscovite	Smectite	Il/Sm Interstratification
EN1	-	-	Trace	-	4	89	1	-	2	-	2
EN2	-	-	2	-	-	87	2	-	2	-	7
EN3	-	-	-	-	14	76	1	-	4	-	5
EN4	-	-	-	-	2	89	2	-	3	1	3
EN5	-	-	-	-	10	72	1	-	8	-	8
ECM6	-	-	-	-	7	84	1	-	2	1	4
ECM7	-	-	-	1	2	93	-	-	1	-	3
ECM8	-	-	3	-	-	33	-	47	-	-	17
ECK9	-	-	-	-	18	73	-	-	1	-	4
ECK10	2	1	-	-	-	70	5	-	15	2	5
ECK11	1	3	-	-	3	61	1	-	16	5	9
ECK12	1	-	1	-	3	80	2	-	5	2	5
ECK13	-	-	-	2	5	93	-	-	1	-	Trace
ECK14	1	1	-	2	-	73	-	7	3	-	13
ECK15	1	-	-	-	1	88	1	-	3	-	5
ECK16	1	-	-	-	5	88	-	-	2	-	4
ECK17	Trace	-	-	Trace	2	93	-	-	1	-	2
FSB18	-	1	-	1	2	75	7	-	3	-	10
FSB19	-	1	-	1	1	68	-	7	5	-	17
FSB20	-	1	-	1	-	69	6	-	6	4	13
FSB21	-	1	1	1	-	71	3	-	6	-	17
FSB22	-	2	-	1	6	61	3	-	15	-	13
FSB23	-	1	-	1	-	73	5	-	5	2	13
FSB24	-	-	-	2	1	74	-	6	1	2	14
FSB25	-	-	-	3	7	79	-	2	7	2	-
FSB26	-	-	6	-	-	52	-	29	-	3	9
FSB27	-	-	-	-	12	79	-	-	4	1	-

In samples from Swaziland, quartz and kaolinite were the dominant minerals identified with significant quantities of muscovite, microcline and plagioclase present in some samples (Table 4). Dominant minerals in the samples from DRC were kaolinite, quartz, anatase, and goethite/hematite (Table 5). Hematite and goethite were present in a few samples from all three countries (Table 3, Table 4 and Table 5). Other minerals which occurred in either trace quantities or constituted <7% of the mineral assemblage included halite (FSB27 and ECM6), calcite (EN1), ilmenite (ECK9), serpentine (FSB27), clinopyroxene (ECK9), and dolomite (ECK11, SZ30). Among the secondary minerals identified with >7% abundance were kaolinite, illite, smectite and illite/smectite interstratification (Table 3, Table 4 and Table 5).

**Table 4:** Mineral assemblage of soil samples from Swaziland.

Sample Code	Mineral Abundance (Wt %)						
	Hematite/ Goethite	Microcline/ Rutile	Plagioclase	Quartz	Kaolinite	Muscovite	Il/Sm Interstratification
SZ28	1	Trace	-	76	23	-	-
SZ29	6	4	22	60	-	-	8
SZ30	2	1	-	47	45	-	-
SZ31	-	2	30	30	20	18	-
SZ32	5	-	-	13	80	-	-
SZ33	-	1	Trace	79	16	3	-
SZ34	-	-	37	1	22	40	-
SZ35	-	10	-	77	11	1	-
SZ36	14	-	-	49	37	-	-
SZ37	-	-	1	-	94	6	-
SZ38	-	8	-	77	13	2	1
SZ39	3	2	-	42	54	-	-

**Table 5:** Mineral assemblage of soil samples from DRC.

Sample	Mineral Abundance (Wt%)												
	Siderite	Halite	Alunite	Gibbsite	Goethite/Hematite	Anatase	Microcline/Rutile	Plagioclase	Quartz	Kaolinite	Mica	Smectite	I/S Interstratification
DRC-1	-	4	-	3	-	2	1	-	35	51	3	2	-
DRC-2	-	-	-	2	-	2	1	1	35	52	3	4	-
DRC-3	-	-	-	-	3	-	-	-	12	82	2	2	-
DRC-4	-	3	-	5	-	2	-	-	29	57	1	2	-
DRC-5	-	-	1	-	3	1	-	1	21	59	14	-	-
DRC-6	-	-	-	2	-	3	-	-	53	43	-	-	-
DRC-7	-	-	1	-	3	2	1	-	22	56	15	-	-
DRC-8	-	-	-	-	-	3	-	-	41	46	10	-	-
DRC-9	6	-	-	-	2	2	-	-	8	82	-	-	-
DRC-10	-	-	-	-	1	-	3	-	67	10	5	-	14
DRC-11	-	-	-	-	-	-	-	-	0	-	-	1	-
DRC-12	4	-	-	-	1	4	-	-	6	86	-	-	-
DRC-13	-	-	-	-	-	3	5	-	57	23	-	4	8
DRC-14	-	-	-	-	2	4	-	-	35	59	-	-	-
DRC-15	-	-	-	-	-	2	-	-	11	87	-	-	-

Whereas the illite/smectite interstratification was the dominant secondary mineral phase identified in samples from SA, kaolinite was more abundant in soils from Swaziland and kaolinite and smectite in samples from DRC (Table 3, Table 4 and Table 5).

### 4.5. Extent of Weathering of Soil Samples

The concentrations of major oxides are used as an indication of the degree of weathering of soils. According to Nesbitt and Young [44], CIA values of 45–55 indicate no weathering. Whereas kaolinite, chlorite, gibbsite, and bohemite have CIA values of 100, smectite and illite have CIA values between the 70–80 range. The mean CIA values for the soil samples were significantly higher in soil samples from DRC (95.4 ± 2.1) than those of Swaziland (91.2 ± 2.4) and South Africa (81.6 ± 1.7) (*p* < 0.05). Similarly, values for CIW of the soils were also higher for samples from DRC than those from Swaziland and South Africa (Table 2).

## 5. Discussion

### 5.1. Granulometric Properties of Soil Samples

Differences in mean contents of sand, silt and clay and mean median diameter of the soil particles were significant between geophagic soils from DRC and those from Swaziland and South Africa (*p* < 0.05). The ranges in volume percent of sand, silt, and clay in these samples are similar to those reported by Aufreiter *et al.* [8], Abraham and Parsons [47], Mahaney *et al.* [48] and Kikouama *et al.* [49] in soil samples ingested from other countries. The results of the particle size analyses depict greater variations in the PSD of geophagic soils from South Africa compared to those from Swaziland and DRC. The variation is also evident in the curvature of particle distribution curves (Figure 2) which are flatter for samples from South Africa (Figure 2a), followed by those from Swaziland (Figure 2b) and then those from DRC (Figure 2c). The PSD curves of the samples also indicate that geophagic soils from South Africa were the most coarse followed by those from Swaziland, and then DRC as is reflected by the values for diameters at the 10^th^ [D(v,0.1)] and 90^th^ [D(v,0.9)] percentiles (Table 1) which are highest for geophagic soils from South Africa and lowest for those from DRC.

**Figure 3:**
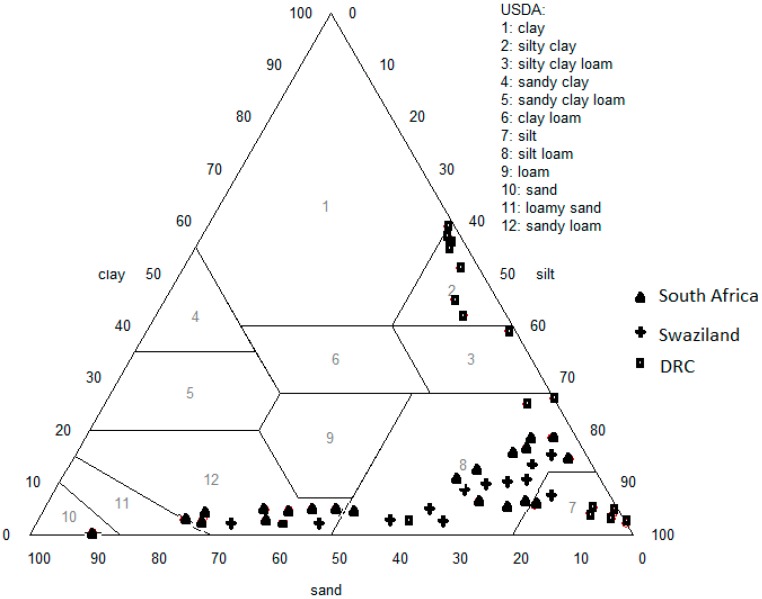
Textural triangle showing the textural classes of the geophagic samples from different countries.

The textural classification of the soil samples places most of the samples from South Africa and Swaziland within the sandy loam and silty loam textural groups whereas the soils from DRC were mostly classified as silt or silty clay (Figure 3). This textural classification of the soils contradicts the belief that soils that are deliberately ingested are clayey in texture. The current processing methods of heating and addition of salts employed by vendors of these geophagic soils would have no influence on the granulometric properties which are determined by the soil particle size distribution. Heating may however decrease the parasite and pathogen load of the samples reducing the potential negative effects of geophagia.

Soil samples from DRC had significantly lower SMD values compared to those from South Africa and Swaziland (*p* < 0.05). The smaller the SMD, the greater the surface area: volume ratio of the samples. The larger surface area in the DRC soil samples implies a higher surface charge density with an increased capacity to sorp cations and toxic compounds from the GIT of the geophagic individual.

The results of the granulometric properties of the soils highlight the differences in granulometric properties of soils ingested by humans. It also indicates that the soils ingested are relatively coarse and vary from one country to another.

### 5.2. Mineralogical Properties of the Soils

Primary minerals constituted the greater percentage of the mineral assemblage of the soils from South Africa and is reflected in the textural properties. The coarse soils from South Africa are predominantly composed of quartz sand, which also explains the high SiO_2_ content from XRF analyses. Quartz content was lower in samples from Swaziland and DRC than in soils from South Africa but there was also a higher amount of secondary minerals in the samples from Swaziland and DRC compared to the samples from South Africa. Primary minerals usually have a lower cation exchange capacity which influences the sorption capacity of the soils. Studies by severance *et al.* [16] have shown that when soils have a high CEC they may scavenge for cations in the GIT upon ingestion.

The content of Fe bearing minerals like hematite, goethite, and siderite was relatively low indicating that the soils may be low in Fe content. This is also reflected in the content of Fe_2_O_3_(t) which was relatively low compared to some ferralsols but higher than has been reported in geophagic soils from Tanzania [50]. Burning or baking of some of the soils by the vendors is not likely to have affected the minerals composition of the samples because heating is done at much lower temperatures than would affect soil mineral transformation. Studies by Richardson [51] documented decomposition of kaolinite at temperatures between 500 °C–700 °C by losing lattice water. Gibbsite alters to amorphous phase at temperatures as low as 200 °C whereas goethite is transformed to hematite at temperatures of approximately 300 °C [52]. Dehydroxylation of most minerals will occur at about 500°C. However, the addition of salt by the vendors would have modified the minerals assemblage of the DRC soils hence the occurrence of halite in some of the samples from DRC.

**Figure 4:**
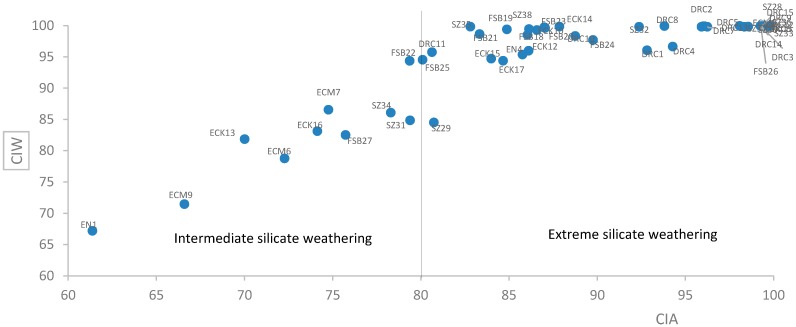
Plot of Chemical Index of Weathering against Chemical Index of Alteration of studied soil samples.

The dominance of primary minerals in the soils from South Africa reflect lower levels of weathering as is evidenced by the lower values of CIA and CIW. The higher values of CIA and CIW obtained for samples from DRC and Swaziland may explain the higher content of clay particles in the samples from these countries. A plot of CIW against CIA of the soil samples (Figure 4) indicate that all but two samples from Swaziland and all soil samples from DRC were extremely weathered compared to those from South Africa.

The CIA is based on the assumption that the dominant process during chemical weathering is degradation of feldspar and the formation of clay minerals [53]. Excessive weathering of the soil results in high clay content and accumulation of silicate clays which may affect several processes in the gut of the individual ingesting the soil. Highly weathered soils are usually depleted of base cations because the process of chemical weathering results in leaching out of most of these cations from the soils.

### 5.3. Major and Trace Element Oxides in Soil Samples

The concentrations of oxides in soils varied but SiO_2_, Al_2_O_3_ and Fe_2_O_3_ had the highest concentration among all the oxides. The siliceous nature of the soils is similar to those reported by Young *et al.* [50] in geophagic soils from Zanzibari, but the content of ferric oxides in geophagic soils from South Africa, Swaziland and DRC were higher. Higher concentration of SiO_2_ in the soils is explained by the dominance of quartz in the samples whereas the presence of Fe-containing minerals including hematite and goethite would explain the observed ferric oxide concentrations. Young *et al.* [50] also found variable and low concentrations of oxides of the major cations Ca, Mg, K and Na in geophagic soils from Zanzibari. The higher values observed for K_2_O among the major oxides especially in the soils from Swaziland and South Africa compared to soils from DRC can be explained by the content of K-feldspars, which were more dominant in the minerals assemblage of samples from Swaziland and South Africa than in soil from DRC. Low concentrations of the oxides of major cations Ca, Mg, K and Na in the soils brings to question the ability of these soils to serve as nutrient supplements for these elements. Baking or burning of the soils is not likely to have any effect on the elemental concentrations in the geophagic soils as heating is usually done at temperatures of below 200 °C, which is not likely to affect the major elements. Soil nutrient elements according to Raison *et al.* [54] have volatilization threshold temperatures that range from 774 °C for potassium to 1962 °C for magnesium. These temperatures are much higher than the temperatures at which these soils are baked or burnt by vendors.

### 5.4. Association of Geophagic Soils

**Figure 5:**
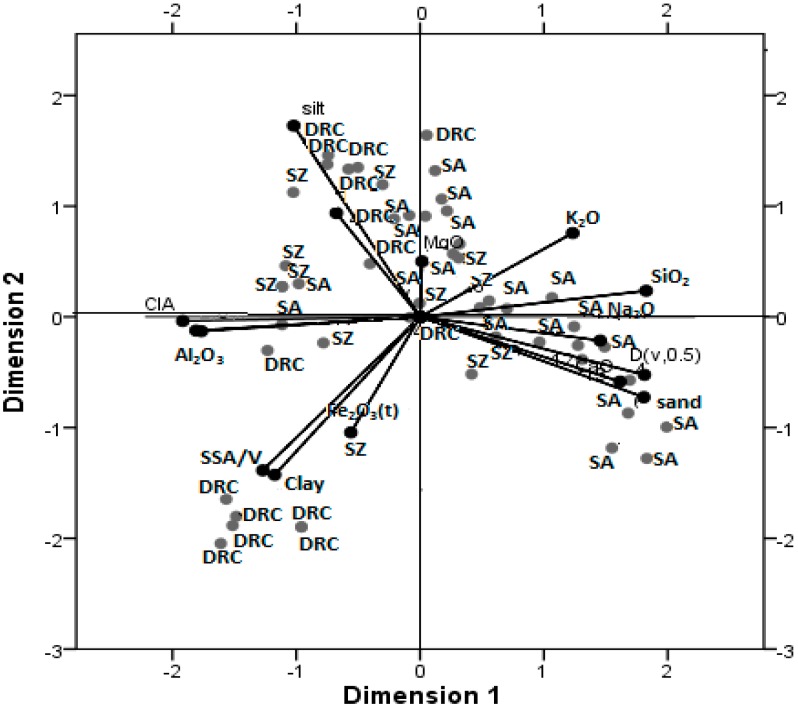
1^st^ and 2^nd^ components of geochemical and granulometric properties of geophagic soil samples.

The properties of soils that have not been influenced by anthropogenic activities show specific relationship with each other. Multivariate analyses of soil properties are sometimes used to understand associations of soil properties to understand any anomalies. Biplots from principal component analyses indicate two main factors contributing 50% of the variance observed in the properties of the soils. A greater percentage of samples from South Africa were associated together with SiO_2_, sand, K_2_O, CaO, MgO positively on the first component whereas samples from DRC associated with silt, clay, Al_2_O_3_, and CIA negatively on the same component. Samples from Swaziland were mainly associating with silt, Na_2_O and MgO positively on the second component (Figure 5).

According to Drew *et al.* [55] and Grunsky and Smee, [56], major element oxides including Al_2_O_3_, Na_2_O, K_2_O, SiO_2_, are associated with feldspathic clays whereas MgO, Fe_2_O_3_, H_2_O, CaO, are usually associated with Fe oxides and Fe-Mg silicate minerals. The inverse relationship observed between Al_2_O_3_ and SiO_2_ can be attributed to weathering of the minerals especially because the DRC samples associated with Al_2_O_3_ contained secondary minerals and had higher CIA values.

The groupings observed in Figure 5 could be explained by the mineralogical composition of the samples and present no anomalies with regards to geochemical properties of soils. The characteristics of the soils do not differ from those of regular soils except for the geophagic soils from DRC which had higher clay content than would normally occur in ordinary soils. Some of the soils from DRC might have been collected from clay deposits explaining the high clay content. However, the associations among soil properties present no anomalies from normal geochemical associations in soil. The associations also indicate that the processing of the soils by vendors through heating has not significantly modified the properties of the geophagic soils.

### 5.5. Possible Health Implication of Ingesting These Soils

Considering the role played by soil textural and mineralogical characteristics in the behavior of soils, inferences on the likely outcome of ingesting soils could be made from knowledge of the granulometric and mineralogical characteristics of a soil. The coarse texture of the soils are likely to present some health threats to the individuals ingesting the soils. Studies on hominid species have shown that medium sand particles (having a diameter ranging from 250 µm–500 µm) cause severe dental damage [32]. Perforation of the sigmoid colon has also been associated with ingestion of coarse-textured soils [57]. The coarse nature of the geophagic soil samples and the wide variation in PSD may require some basic beneficiation processes to reduce the amount of coarse-textured particles present in order to reduce potential health threats to the individual ingesting them. Removal of the coarse textured particles through sieving may improve the quality of the soil while reducing these potential health threats. An increase in smaller sized fraction would provide certain benefits to the individual including an increase in the bactericidal properties of the soils [58] while protecting the gastrointestinal epithelium through the formation of cross linkages with glycoprotein in the intestinal mucosa [59]. Increased clay content may also increase the sorption capacity of the soil affecting the amount of nutrients that are assimilated by the individual from the GIT.

The ingestion of soil for treatment of ailments, food detoxification, and alleviation of gastrointestinal disorders, such as diarrhea is related to the soils ability to sorp materials from the GIT. The mineral composition of the soils studied, especially those from South Africa, and Swaziland coupled with their low clay content and low surface area implies that these soils have a low sorption capacity. This is due to the dominance of kaolinite in their mineral assemblage which has a lower CEC range of 3–15 meq/100 g compared to other clay minerals like illite (24–40 meq/100 g), montmorillonite (80–150 meq/100 g) and vermiculite (80–150 meq/100 g). Kikouama *et al.* [49] have reported that ingesting soils with high clay content could result in protection of the GIT because high specific surface area implies an increased capacity to adsorb toxic compounds from the GIT of the individual. Important nutrient cations including Fe, and Ca could also be sorped from the GIT resulting in the individual displaying deficiency symptoms of these nutrient elements [60]. It is however not known what percentage of clay in soils is needed for a soil to have these therapeutic. Future studies are therefore needed to fill this information gap. The soils studied are not likely to contribute to cation deficiencies in the individual ingesting the soil because of their low sorption capacity.

One of the reasons used to justify geophagia is the supplementation of elements including Ca, Fe, and Mn among others [1,6]. The extent of weathering that has taken place in these samples and the low values of the oxide concentrations of these elements in the samples indicate that these soils may not be suitable as a supplement for these elements in the individual ingesting the soils. Studies on bioavailability of these elements in the GIT are however necessary to determine which percentage of the total concentration is actually available for assimilation upon ingestion. In studies carried out by Seim *et al.* [23] and Pebsworth *et al.* [24] on geophagic soils, it was revealed that higher concentrations of Fe in geophagic soils does not necessarily imply high bioavailability. Their study also showed that Fe content is not necessarily the reason why baboons for example ingest soils as the preferred soil had lower Fe content than other geophagic soils. It is unlikely that Fe supplementation is the main factor that motivates geophagy and even if that were the case in other soils, the possibility of the soils in this study serving as Fe supplements may be low considering the low ferric oxide content and low amounts of Fe-bearing minerals in the soils, and the fact that not all the Fe present in the soil is available for uptake. Hooda and Henry [17] reported 29–79% bioavailability of Fe in soils from Uganda, Tanzania, Turkey, and India. Studies carried out by Abrahams *et al.* [61] for example reported that Pb bioaccessibility in geophagic soils from United Kingdom vary between 3–83% whereas that of Hg varies with its chemical form.

Studies by Severance *et al.* [16] and Brouillard and Rateau, [18] have also indicated that increased deficiency of certain elements in geophagic individuals may occur as a result of the high cation exchange capacity of the soils which increases their ability to sorp nutrients from the GIT. The soils in this study are not expected to contribute to a deficiency of these elements in the individual ingesting them as the CEC of the samples are relatively low.

## 6. Conclusions

This study has revealed a wide variation in the granulometric and mineralogical properties as well as the concentrations of major and trace element concentrations of soils that are ingested. The soils from Swaziland and South Africa by virtue of their granulometric and mineralogical properties are not likely to achieve the various objectives which have been used to justify geophagia. Extensive weathering of the geophagic samples from DRC also reduces their potential as supplements of various nutrients including Fe, Ca, and K. To reduce the health risk that may be associated with the ingestion of coarse textured soils, basic soil beneficiation processes aimed at reducing the coarse particles would be necessary as this fraction has been implicated as a major threat to a practicing geophagist.

## References

[1] WHO (1996). Trace Elements in Human Nutrition and Health.

[2] Songca S.P., Ngole V.M., Ekosse G.E., De Jager L. (2010). Demographic characteristics associated with consumption of geophagic clays among ethnic groups in the Free State and Limpopo provinces. Indilinga: Afr. J. Indigen. Knowl. Syst..

[3] Henry J.M., Cring F.D., Brevik E.C., Burgess L.C. (2013). Geophagy: An Anthropological perspective. Soils and Human Health.

[4] Dean J.R., Deary M.E., Gbefa B.K., Scott W.C. (2004). Characterization and analysis of persistent organic pollutants and major, minor and trace elements in calabash chalk. Chemosphere.

[5] Jones R.L., Hanson H.C. (1985). Mineral Licks, Geophagy and Biogeochemistry of North American Ungulates.

[6] Johns T., Duquette M. (1991). Detoxification and mineral supplementation as functions of geophagy. Am. J. Clin. Nutr..

[7] Mahaney W.C., Hancock V.G.R., Aufreiter S., Huffman M.A. (1996). Geochemistry and clay mineralogy of termite mound soil and the role of geophagy in chimpanzees of the Mahale Mountains, Tanzania. Primates.

[8] Aufreiter S., Hancock R.G.V., Mahaney W.C., Stambolic-Robb A., Sanmugadas K. (1997). Geochemistry and mineralogy of soils eaten by humans. Int. J. Food Sci. Nutr..

[9] Olivier M.A. (1997). Soil and human health: A review. Eur. J. Soil Sci..

[10] Hooda P.S. Soil ingestion affects the potential bioavailability of Cu, Mn, and Zn. Proceedings of the 7th International Conference on the Biogeochemistry of Trace Elements.

[11] Wilson M.J. (2003). Clay mineralogical and related characteristics of geophagic materials. J. Chem. Ecol..

[12] Dominy N.J., Davoust E., Minekus M. (2004). Adaptive function of soil consumption: An *in vitro* study modelling the human stomach and small intestine. J. Exp. Biol..

[13] Gilardi J.D., Duffey S.S., Munn C.A., Tell L.A. (1999). Biochemical functions of geophagy in parrots: Detoxification of dietary toxins and cytoprotective effects. J. Chem. Ecol..

[14] Wang P., Afriyie-Gyawu E., Tang Y., Johnson N.M., Xu L., Tang L., Huebner H.J., Ankrah N.A., Ofori-Adjei D., Ellis W., Jolly P.E., Williams J.H., Wang J.S., Phillips T.D. (2008). NovaSil clay intervention in Ghanaians at high risk for aflatoxicosis: II. Reduction in biomarkers of aflatoxin exposure in blood and urine. Food Addit. Contam. Part A Chem. Anal. Control Exp. Risk Assess..

[15] Afriyie-Gyawu E., Ankrah N.A., Huebner H.J., Ofosuhene M., Kumi J., Johnson N.M., Tang L., Xu L., Jolly P.E., Ellis W.O., Ofori-Adjei D., Williams J.H., Wang J.S., Phillips T.D. (2008). Novasil clay intervention in Ghanaina at risk of aflatoxins: I. Study design and clinical outcomes. Food Addit. Contam. Part A Chem. Anal. Control Exp. Risk Assess..

[16] Severance H.W., Holt T., Patrone N.A., Chapman L. (1998). Profound muscle weakness and hypokalemia due to clay ingestion. S. Med. J..

[17] Hooda P., Henry J., MacClancy J., Henry J., MacBeth H. (2007). Geophagia and human nutrition. Consuming the Inedible: Neglected Dimensions of Food Choice.

[18] Brouillard M.Y., Rateau J.G. (1989). Smectitie and kaolin on bacterial enterotoxins. Gastroen. Clin. Biol..

[19] Glickman L.T., Camara A.O., Glickman N.W., Mccabe G.P. (1999). Nematode intestinal parasite of children in rural Guinea, Africa: Prevalence and relationship to geophagia. Int. J. Epidemiol..

[20] Saathoff E., Olsen A., Kvalsvig J.D., Geissler P.W. (2002). Geophagy and its association with geohelminth infections in rural schoolchildren from northern KwaZulu Natal—South Africa. Trans. Roy. Soc. Trop. Med. Hyg..

[21] Kawai K., Saathoff E., Antelman G., Msamanga G., Fawzi W.W. (2009). Geophagy (soil-eating) in relation to anaemia and helminth infection among HIV-infected pregnant women in Tanzania. Am. J. Trop. Med. Hyg..

[22] Shigova W., Moturi W. (2009). Geophagia as a risk factor for diarrhea. J. Infect. Develop. Countries.

[23] Seim G.L., Ahn C.I., Bodis M.S., Luwedde F., Miller D.D., Hillier S., Tako E., Glahn R.P., Young S.L. (2013). Bioavailability of iron in geophagic earths and clay minerals, and their effect on dietary iron absorption using an *in vitro* digestion/Caco-2 cell model. Food Funct..

[24] Pebsworth P.A., Seim G.L., Huffman M.A., Glahn R.P., Tako F., Young S.L. (2013). Soil consumed by Chacma baboons is low in bioavailable iron and high in clay. J. Chem. Ecol..

[25] Ekosse G.E., de Jager L., Ngole V. (2010). Traditional mining and mineralogy of geophagic clays from Limpopo and Free State provinces, South Africa. Afr. J. Biotechnol..

[26] Ngole V.M., Ekosse G.E. (2012). Physico-chemistry, mineralogy and geochemistry of geophagic clayey soils from Eastern Cape, South Africa, and their nutrient bioaccessibility. J. Sci. Res. Essays.

[27] Abrahams P.W. (1997). Geophagy (soil consumption) and iron supplementation in Uganda. Trop. Med. Int. Health.

[28] Mahaney W., Hancock R.G.V., Inoue M. (1993). Geochemistry and clay mineralogy of soils eaten by Japanese macaques. Primates.

[29] Kutalek R., Wewalka G., Gundacker C., Auer H., Wilson J., Haluza D., Huhulescu S., Hillier S., Sager M., Prinz A. (2010). Geophagy and potential health implications: Geohelminths, microbes and heavy metals. Trans. Roy. Soc. Trop. Med. Hyg..

[30] Callahan G.N. (2003). Eating dirt. Emerg. Infect. Dis..

[31] Harvey W.J.P., Dexter P.B., Darton-Hill I. (2000). The impact of consuming iron from non-food sources on iron status in developing countries. Public Health Nutr..

[32] King T., Andrews P., Boz B. (1999). Effect of taphonomic processes on dental microwear. Am. J. Phys. Anthropol..

[33] Prince R.J., Luoba A.I., Adhiambo P., Ng’uono J., Geissler P.W. (1999). Geophagy is common among Luo woman in western Kenya. Trans. Roy. Soc. Trop. Med. Hyg..

[34] Hunter J.M., de Kleine R. (1984). Geophagy in Central America. Geogr. Rev..

[35] Remmelzwaal A., Masuku B.S. (1994). Characterization and Correlation of the Soils of Swaziland. Land Use Planning for Rational Utilization of Land and Water Resources Project SWA 89/001.

[36] Kasongo R.K., Verdoodt A.P., Kanyankagote P., Baert G., Van Ranst E. (2011). Coffee waste as an alternative fertilizer with soil improving properties for sandy soils in humid tropical environments. Soil Use Manage..

[37] Mandiringana O.T., Mnkeni P.N.S., Mkile Z., van Averbeke W., van Ranst E., Verplancke H. (2005). Minerology and fertility status of selected soils of the Eastern Cape Province, South Africa. Commun. Soil Sci. Plant Anal..

[38] Van Reeuwijk L.P. (2002). Procedures for Soil Analysis, Technical Paper, No. 9.

[39] Council for Geosciences Guide to the Services of the CGS Analytical Laboratory. http://196.33.85.14/cgs_inter/images/stories/Lab_Guide/Services_of_the_CGS_Analytical_Laboratory.pdf.

[40] Brime C. (1985). The accuracy of X-ray diffraction methods for determining mineral mixtures. Miner. Mag..

[41] Bish D.L., Reynolds R.C., Bish D.L., Post J.E. (1989). Sample preparation for X-ray diffraction. Reviews in Mineralogy: Modern Powder Diffraction 20.

[42] Moore D.M., Reynolds R.C. (1997). X-ray Diffraction and the Identification and Analysis of Clay Minerals.

[43] Fitton G., Gill R. (1997). X-ray fluorescence spectrometry. Modern Analytical Geochemistry: An Introduction to Quantitative Chemical Analysis Techniques for Earth, Environmental and Material Sciences.

[44] Nesbitt H.W., Young G.M. (1982). Early Proterozoic climates and plate motions inferred from major element chemistry of luttites. Nature.

[45] Harnois L. (1988). The CIW index: A new chemical index of weathering. Sediment. Geol..

[46] Zhou D., Chang T., Davis J.C. (1983). Dual Extraction of R-Mode and Q-Mode Factor Solutions. Math. Geol..

[47] Abrahams P.W., Parsons J.A. (1997). Geophagy in the Tropics: An Appraisal of Three Geophagical Materials. Environ. Geochem. Health.

[48] Mahaney W.C., Milner M.W., Mulyono H.S., Hancock R.G.V., Aufreiter S., Reich M., Wink M. (2000). Mineral and chemical analyses of soils eaten by humans in Indonesia. Int. J. Environ. Health.

[49] Kikouama O.J.R., Konan K.L., Bonnet J.P., Baldé L., Yagoubi N. (2009). Physicochemical characterization of edible clays and release of trace elements. Appl. Clay Sci..

[50] Young S.L., Wilson M.J., Hillier S., Delbos E., Ali S.M., Stoltzfus R.J. (2010). Differences and Commonalities in Physical, Chemical and Mineralogical Properties of Zanzibari Geophagic Soils. J. Chem. Ecol..

[51] Richardson H.M., Brown G. (1972). Phase changes which occur on heating kaolin clays. The X-Ray Identification and Crystal Structures of Clay Minerals.

[52] Cornell R.M., Chwertmann U. (1996). The Iron Oxides: Structure, Properties, Reactions, Occurence and Uses.

[53] Goldberg K., Humayun M. (2010). The applicability of the Chemical Index of Alteration as a paleoclimatic indicator: An example from the Permian of the Paraná Basin, Brazil. Paleogeography, Paleoclimate, Paleoecol..

[54] Raison R.J., Khanna P.K., Woods P.V. (1985). Mechanisms of element transfer to the atmosphere during vegetation fires. Can. J. Forest Res..

[55] Drew L.J., Grunsky E.C., David M., Sutphin D.M., Woodruff L.G. (2010). Multivariate analysis of the geochemistry and mineralogy of soils along two continental-scale transects in North America. Sci. Total Environ..

[56] Grunsky E.C., Smee B.W. (2003). Enhancements in the interpretation of geochemical data using multivariate methods and digital topography. Can. Inst. Min. Met..

[57] Lohn J.W.G., Austin R.C.T., Winslet M.C. (2000). Unusual causes of small-bowel obstruction. J. Roy. Soc. Med..

[58] Haydel S.E., Remenih C.M., Williams L.B. (2008). Broad-spectrum *in vitro* antibacterial activities of clay minerals against antibiotic-susceptible and antibiotic-resistant bacterial Pathogens. J. Antimicrob. Chemother..

[59] Rateau J.G., Morgant G., Droy-Priot M.T., Parier J.L. (1982). A histological, enzymatic and water-electrolyte study of the action of smectite, a mucoprotective clay, on experimental infectious diarrhoea in the rabbit. Cur. Med. Res. Opin..

[60] Brevik E.C., Burgess L.C., Brevik E.C., Burgess L.C. (2013). Soils and human health: An overview. Soils and Human Health.

[61] Abrahams P.W., Follansbee M.H., Hunt A., Smith B., Wragg J. (2006). Iron nutrition and possible lead toxicity: an appraisal of geophagy undertaken by pregnant women of UK Asian communities. Appl. Geochem..

